# CRISPR Associated Diversity within a Population of *Sulfolobus islandicus*

**DOI:** 10.1371/journal.pone.0012988

**Published:** 2010-09-28

**Authors:** Nicole L. Held, Alfa Herrera, Hinsby Cadillo-Quiroz, Rachel J. Whitaker

**Affiliations:** Department of Microbiology, University of Illinois, Urbana-Champaign, Urbana, Illinois, United States of America; Columbia University, United States of America

## Abstract

**Background:**

Predator-prey models for virus-host interactions predict that viruses will cause oscillations of microbial host densities due to an arms race between resistance and virulence. A new form of microbial resistance, CRISPRs (clustered regularly interspaced short palindromic repeats) are a rapidly evolving, sequence-specific immunity mechanism in which a short piece of invading viral DNA is inserted into the host's chromosome, thereby rendering the host resistant to further infection. Few studies have linked this form of resistance to population dynamics in natural microbial populations.

**Methodology/Principal Findings:**

We examined sequence diversity in 39 strains of the archeaon *Sulfolobus islandicus* from a single, isolated hot spring from Kamchatka, Russia to determine the effects of CRISPR immunity on microbial population dynamics. First, multiple housekeeping genetic markers identify a large clonal group of identical genotypes coexisting with a diverse set of rare genotypes. Second, the sequence-specific CRISPR spacer arrays split the large group of isolates into two very different groups and reveal extensive diversity and no evidence for dominance of a single clone within the population.

**Conclusions/Significance:**

The evenness of resistance genotypes found within this population of *S. islandicus* is indicative of a lack of strain dominance, in contrast to the prediction for a resistant strain in a simple predator-prey interaction. Based on evidence for the independent acquisition of resistant sequences, we hypothesize that CRISPR mediated clonal interference between resistant strains promotes and maintains diversity in this natural population.

## Introduction

Virus-host interactions are a prominent driver of microbial diversity in natural environments. The simplest models describe these interactions through predator-prey dynamics which result in temporal fluctuations in strain dominance similar to ecological Lotka-Volterra models [1]–[3]. Without a cost to resistance and in a homogenous environment, these models predict that populations exhibit oscillations in host abundance in their arms race with viral predators [4]–[8]. Such models have been verified experimentally with microbial populations in chemostats [7]. Also, using community genomics of host and virus, Rodriguez-Brito et al. demonstrated change in the viral populations through time suggestive of these dynamics [9].

Oscillations of different species' abundance are theorized to be damped by fitness trade-offs associated with the physiological costs of viral resistance, resulting in a stable level of diversity of coexisting strains within a population at any one time [10]. This is generalized from the kill-the-winner model, where the winner has a competitive advantage in resource utilization, but is susceptible to predation [11]. For example, the theoretical predictions of Weitz et al. find that host and virus can coexist and diversify in a homogenous culture with a single resource due to variation in the trade-offs associated with phage resistance and viral virulence [10]. These dynamics have been demonstrated experimentally in chemostat cultures of *Cellulophaga baltica* infected with two virulent bacteriophages [12]. In this study, Middelboe et al. showed that, upon infection by phage, the coexisting lineages of *Flavobacterium* diversified physiologically and in phage susceptibility to a panel of phages. In addition, Lennon et al. demonstrated variation in the cost of resistance that could result in a stable level of diversity within a population that is higher than would be predicted if populations were evolving through clonal competition for resources in the absence of viral predation [13]. It has recently been suggested that the genetic source of these variable resistance profiles is phage receptor diversity provided by highly variable, rapidly evolving regions of microbial genomes (genomic islands) [6]. Diversity is further promoted as these dynamics occur in spatially structured populations of hosts and viruses where coevolutionary dynamics allow diversity to persist on a larger scale [14]. Viral predation thereby provides the (non-neutral) mechanism maintaining microbial diversity and provides a solution to the apparent “paradox of the plankton” where seemingly redundant organisms coexist [15].

Predictions from theoretical and experimental studies of virus-host interactions have been challenging to study in wild populations because establishing linkage between genotype and resistance phenotype is difficult using culture independent molecular tools [16]. The recently discovered sequence based CRISPR (clustered regularly interspaced short palindromic repeats) system provides the means to examine virus-host interactions in natural populations using molecular tools. CRISPRs are a microbial system discovered to provide immunity to viruses in *Streptococcus thermophilus* [17] and prevent conjugative transfer of plasmids in *Staphylococcus epidermidis* [18]. Sequence specific resistance, conferred by short DNA spacer sequences on the host chromosome and separated by repeat sequences of similar length, have been shown to match extracellular elements such as viruses and plasmids [19]–[21]. New spacers are incorporated into the genome at one end of the locus, the leader end, with the other end of the locus, the trailer end, representing the oldest spacers in the locus. As with other forms of adaptive immunity, notably that found in humans and other jawed vertebrates, CRISPRs are combinatorial and rapidly evolving [17], [22]–[24].

To examine the effects of CRISPR immunity on population dynamics, Tyson and Banfield reconstructed CRISPR loci from two different populations of one species of *Leptospirilum* group II from acid mine drainage [25]. This study observed that the group of spacers at the trailer end of the locus was generally in conserved order (with some spacer loss) in both populations. Spacers at the middle of the locus were population specific, and towards the leader end of the locus the spacers became strain specific. This is consistent with oscillations in clone abundance caused by a selective sweep of a clone that acquired resistance through a specific spacer sequence, seen by shared spacers at the trailer end of the locus. In contrast, in a more complex microbial mat, Heidelberg et al. rarely saw the same spacer twice, and not necessarily in the same CRISPR spacer context, and therefore were unable to specifically assess the virus-host dynamics of the system [26].

In order to understand the ways in which virus-host interactions mediated by CRISPRs affect population dynamics, it is necessary to link signatures of resistance among coexisting strains to genotypic variation within a population. Analysis of natural population dynamics at a strain-specific level is needed to test predictions of current models about the way that virus-host interactions affect population dynamics [27]. We investigate the diversity present in a single population of *S. islandicus* from a hot spring in the Mutnovsky Volcano region of Kamchatka, Russia. We use multi-locus sequence analysis (MLSA) from a set of core housekeeping genes present in *S. islandicus* to determine overall host diversity and compare this to the diversity identified in CRISPR spacer sequences from each isolate.

## Results

### Relationships among strains by MLSA

**Figure 1:**
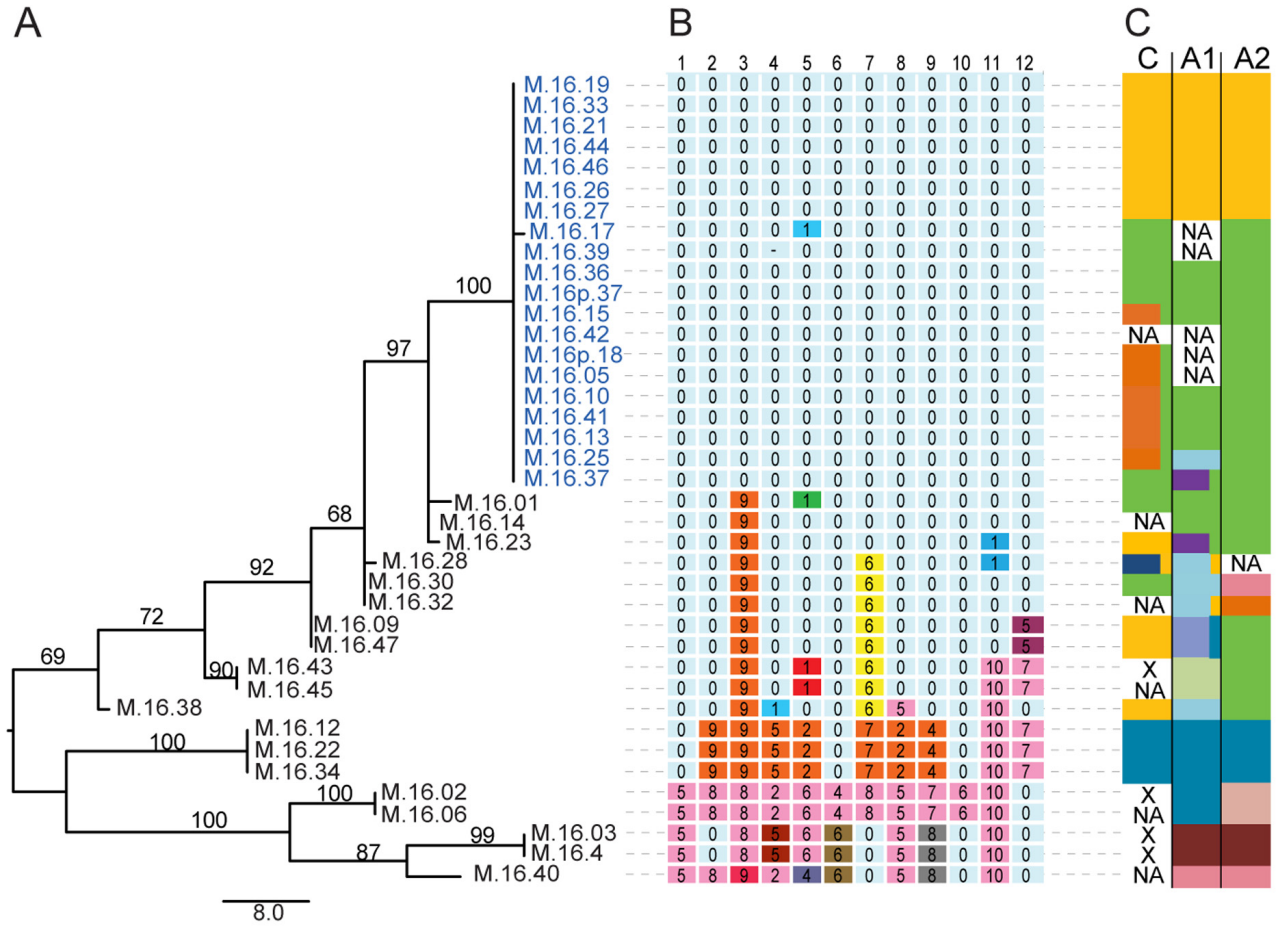
Core gene phylogeny and MLSA allelic profiles compared to CRISPR spacer types. (A) A Maximum Parsimony phylogeny of a concatenated nucleotide alignment of 12 loci (6684 bp) from 39 *S. islandicus* isolates from a single hot spring in Mutnovsky. Scale bar represents eight nucleotide changes. Numbers above branches represent bootstrap support from 1000 replicates. The large group of strains with nearly identical MLSA sequences at core gene loci is highlighted in blue. (B) The allelic profiles of MLSA loci show the number of SNPs in comparison to strain M.16.19, and the background color in each cell indicates the allele type for each locus. (C) The three colored summary bars to the right of the allelic profiles indicate ancestral groupings of each CRISPR locus by shared spacers as in Figure 2. ‘X’ indicates a CRISPR locus is not present and ‘NA’ indicates that a locus could not be sequenced.

Figure 1A shows the Maximum Parsimony phylogeny constructed from the concatenated MLSA data from 12 variable core loci (6684 bp in total) among 39 strains of *Sulfolobus islandicus* from a single hot spring in the Mutnovsky Volcano region of Kamchatka, Russia. As shown in Figure 1B, and has been demonstrated previously [28], this population contains an epidemic population structure [29] in which one dominant genotype (blue names in Figure 1A: 49% of clones) coexists with rare recombinant types containing different combinations of rare alleles. We previously hypothesized that this dominant clone results from a clonal expansion of one type possibly due to viral resistance. Rates of recombination, estimated with 12 new loci using ClonalFrame [30], are close to previous reports [28] using five loci with a recombination to mutation ratio (r/m) of 3.8. Rarefaction curves of the MLSA genotypes (Figure S1A) demonstrate that when OTUs are binned at 0.01% divergence (one SNP per 1000 bp), the diversity of *S. islandicus* in this spring has been well sampled with the 39 strains described here. Chao1 richness is estimated to be 20 OTUs when each individual is unique (one OTU at 0.01% divergence) [31].

**Figure 2:**
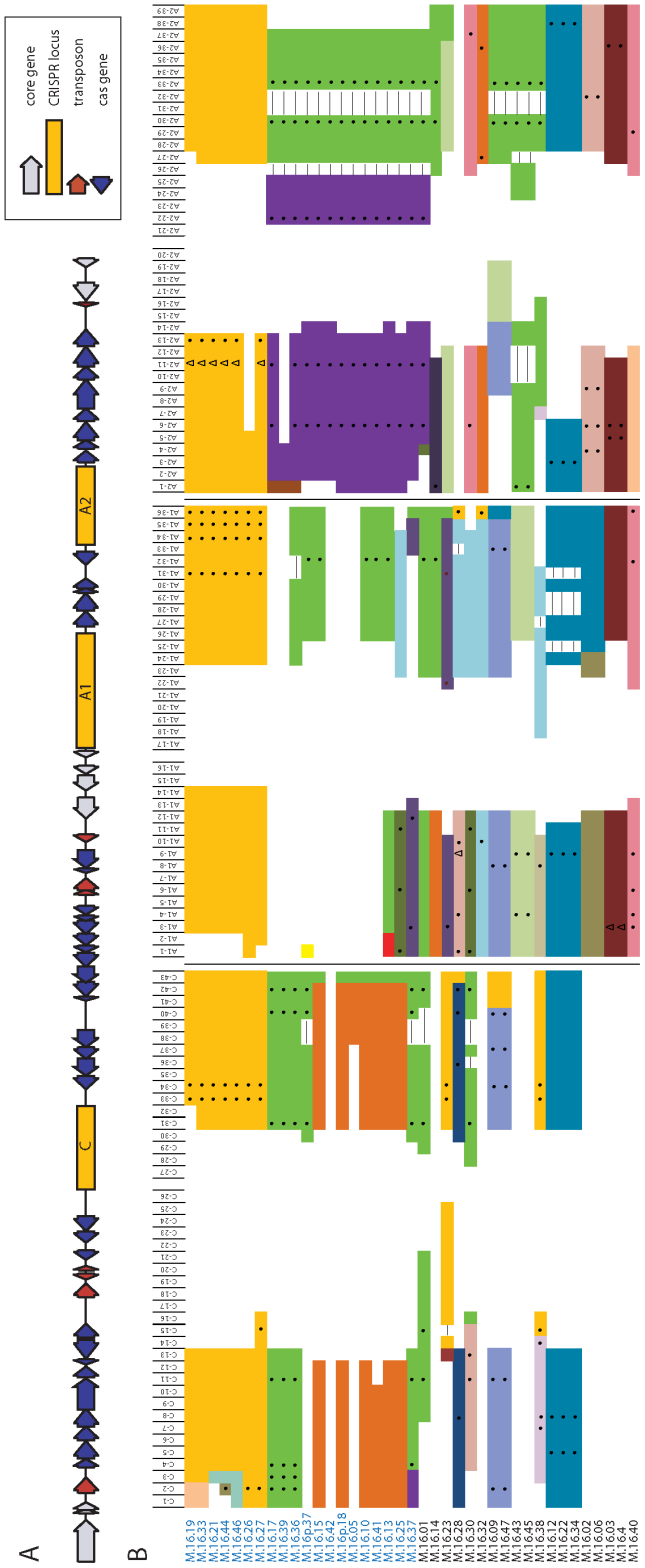
Summary of the CRISPR spacers end sequences in 39 *S. islandicus* strains. (A) A schematic of the CRISPR region of reference genome M.16.27 [33]. Rectangles represent each CRISPR repeat-spacer locus. Core and CRISPR associated (cas) genes are indicated by grey and blue arrows respectively. A red arrow indicates a transposon insertion. The reference genome M.16.27's CRISPR loci are named on the CRISPRdb website [50] as NC_012632_1 (C), NC_012632_1 (A1), and NC_012632_1 (A2). The reference genome M.16.4's CRISPR loci are named NC_012726_1 (A1) and NC_012726_1 (A2). (B) The color-coded CRISPR spacer arrays from left to right are the C locus, the A1 locus, and the A2 locus, as in (A). The *S. islandicus* strains are listed from top to bottom in the same order as in Figure 1. Each box represents a CRISPR spacer, with the spacer positions numbered at the top of the column. The leader end spacers are oriented on the left of each locus while the trailer end spacers are oriented on the right of each locus. Identical spacers in the same spacer context are vertically aligned and given the same color in the column of boxes. White boxes represent missing data and a line through a white box indicates a gap. • in boxes represents independent acquisitions of the same virus or plasmid and Δ represents spacers that match a different part of the same virus.

### Diversity of sequences from CRISPR loci

*S. islandicus* from the Mutnovsky population have up to three CRISPR loci (named C, A1, and A2, see Figure 2A) that encode a sequence based history of interactions between *S. islandicus* and mobile elements such as viruses and plasmids [32]. Figure 2B shows the leader and trailer end sequences from these three loci from 37 new strains and two previously sequenced strains from the M16 hot spring [33]. In total, we sequenced 2374 new CRISPR spacers, with 756 unique spacer sequences of average length 39 bp. Unlike the MLSA data, the rarefaction curve of the coded CRISPR spacer arrays (each spacer represents a single character) shows that the diversity at the CRISPR loci is undersampled with 39 strains from a single hot spring (Figure S1B). Chao1 richness is estimated to be 10 times that estimated for MLSA if every difference is considered unique and is very likely a dramatic underestimation of diversity due to undersampling [31].

Several loci could not be amplified despite development of eight new primer sequences (Figure S2) because of the diversity of sequences surrounding loci of closely related strains. In several cases, failure to amplify loci resulted from the loss of the C locus, as confirmed by genome sequencing and southern hybridizations using the repeat sequence from that locus as a probe (data not shown). At the A1 and A2 loci we were unable to determine whether sequence divergence or loss of the locus prevented amplification, because probes with the A sequence bind to both loci in southern blots. Nevertheless, the presence of these loci in sequenced isolates that failed to amplify suggests sequence divergence rather than loss of these loci by members of this population. These data demonstrate the high level of diversity within this system that appears not only in the spacer sequences but in surrounding genes involved in the CRISPR system as well.

In this population of *S. islandicus*, as has been observed in other studies, the leader ends of all three CRISPR loci are more variable than the trailer ends [17], [24], [25]. Many isolates share the same spacers as another isolate throughout the locus except for the leader-most spacers, likely due to the two isolates sharing a common ancestor at that locus (Figure 2B). As has been experimentally demonstrated for bacterial species (*Streptococcus* sp.), new spacer sequences are added at the leader end in response to invasion of mobile elements [17]. If CRISPR addition occurs in *Sulfolobus* as it does in *Streptococcus,* the variability observed at the leader end is likely to have resulted from recent interactions of *S. islandicus* with viruses or plasmids and indicates that these loci are actively acquiring resistance in this population. For several pairs of isolates, the only remaining evidence of shared spacer sequences in the same position are the very first conserved spacer at the trailer end of the locus (indicated in Figure 1C by the dual-color in the summary bar on the right), demonstrating probable ancestry followed by divergence. Although there is striking diversity in the CRISPR spacer arrays among the 39 isolates, every individual is not unique.

In addition to sequence variation, we also observed variation in CRISPR loci in the *S. islandicus* population that results from spacer loss. Loss is identified by comparing two isolates with the same set of spacers in the same order on either side of a gap in the spacer alignment [21], [25], [34]. *S. islandicus* isolates show evidence of spacer loss, both individual and in sets of up to five spacers, with two being the average size of consecutively lost spacers (as is shown in Figure 2). We tested whether the variability of spacers at the leader end of the locus could actually result from loss of spacers at the leader end. If this were the case, we would expect to see spacers from the leader end of a locus in one strain match those from the middle of a locus in another strain. In the subset of fully sequenced isolates, we were able to search for leader end spacers from other isolates located internally in the fully sequenced loci and did not find any matches, indicating that the leader end sequences are truly unique and result from independent spacer acquisition (data not shown).

Of the 756 unique spacers, only 50 have significant (E*<*0.001) BLAST matches to a database of *Sulfolobus* genomes, viruses, and plasmids. The majority (87%) of these match viruses; 22% of total matches are to viruses integrated into *S. islandicus* genomes and 51% of total matches are to SSV (*Sulfolobus* spindle-shaped virus) sequences, a non-lytic virus that has been isolated from around the world, with many sequenced isolates. The rest of the matches (less than 7% each) are to plasmids and other *Sulfolobus* genomes or other integrated elements within them. As we have shown previously, we do not see spacers that match 100% to a portion of the same genome in which the spacers are located, as is evident in the fully sequenced subset of isolates from this population [32].

### Population structure defined by CRISPR sequences

As shown in Figure 1, the relationships among strains based on CRISPR sequences are very different from those observed by MLSA. The summary of CRISPR locus types (Figure 1C) split the large group of apparently identical MLSA genotypes, hypothesized to represent the epidemic rise in frequency of a single clone, into two groups of isolates with no recognizable evidence of ancestry. Furthermore, members of each of these two new groups of isolates share apparently ancient ancestry with the more divergent rare types observed through MLSA analysis, as is evidenced by their sharing spacer sequences at their trailer ends. As with the MLSA, Figure 1C shows rare recombinant combinations of CRISPR alleles indicating that recombination is also occurring among these loci.

**Figure 3:**
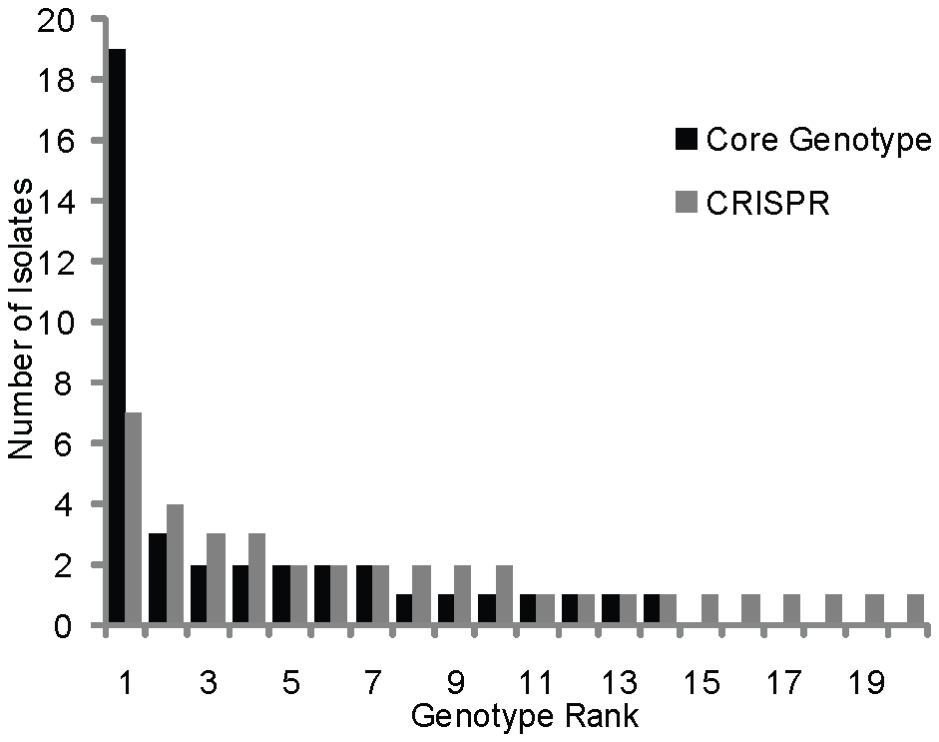
Genotype rank abundance graph of concatenated core and CRISPR end sequences. A genotype rank abundance graph with strains grouped by MLSA core genotypes (black) and CRISPR spacers (grey, by ancestral groupings, as in Figure 1). Groups were ranked by the number of isolates in each and plotted from largest (left) to smallest (right).

Figure 3 shows the difference in population structure within a hot spring based on MLSA and CRISPR sequences. The MLSA core genotype category shows a population structure in which there are a few dominant types, indicating some evidence of selective sweeps in the history of the population. However, contrary to data from metagenomic analyses of microbial diversity, even at their most conserved (trailer end), the CRISPR sequences show evidence for a diversity of coexisting genotypes. Although there are several groups of strains with multiple representatives, CRISPR sequences show no evidence of a selective sweep as would have been predicted based on MLSA data and on theoretical predictions about the rise in frequency of strains resistant to viral infection [3].

### Independent acquisition of CRISPR spacers

**Figure 4:**
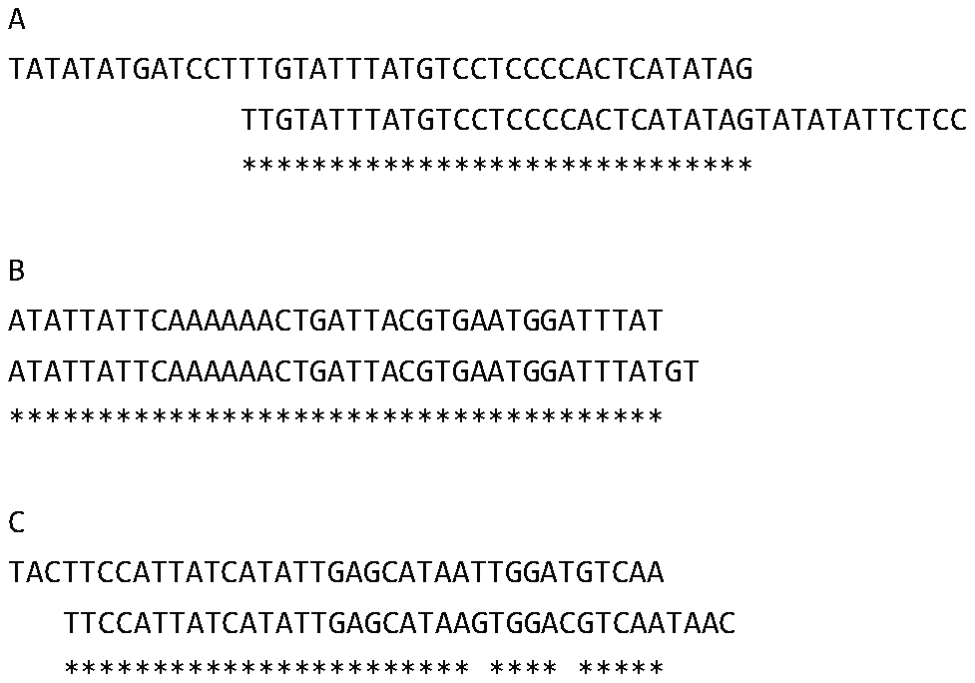
Examples of signatures of independent spacer acquisitions. (A) Spacer pair number 5 from Table S1 shows an example of an offset match with no single nucleotide polymorphisms (SNPs). (B) Spacer pair number 18 from Table S1 shows an example of a match of different length spacers. (C) Spacer pair number 31 from Table S1 shows an example of an offset spacer match with two SNPs. * indicates a shared base between the two spacers. All matches are listed in Table S1.

We compared CRISPR spacer sequences to one another to test for evidence of independent acquisition of spacers to the same virus by different coexisting strains. There are 41 pairs of spacers that match one another at least 88% over a length of at least 17 nt that are not related to one another ancestrally, i.e. in the same context in the locus (spacers that match other spacers are indicated in Figure 2 by dots and are listed in Table S1). Most of these are not identical spacers, but rather are offset (37 out of 41 pairs) as is shown in Figure 4. The incomplete overlap of each spacer, in addition to its unique position in the spacer array, indicates that it represents an independent acquisition of a spacer from nearly the same location in the same virus or plasmid. One pair of consecutive spacers match, between two strains, 100% in sequence over 100% of the length in a different leader end context (M.16.27 C-15 and C-16 and M.16.38 C-15 and C-16, Figure 2). Because it is unlikely that two consecutive spacers that match exactly between two strains were independently acquired by each strain, we have excluded these from our analysis of independent acquisition.

18 (44%) of the independently acquired matching spacer pairs are located on the leader ends of loci in both strains, which indicates that both spacers were recent acquisitions of an element that was present in the spring at that time. Pairs with both trailer end spacers make up 27% of the matches and mixed leader and trailer matches make up the remaining 29%. We did not observe a locus preference for spacers as has been suggested previously [35]. Of the 41 pairs of independently acquired spacers that match one another, only one pair is from strains that have similar CRISPR spacer arrays. The rest are between divergent strains with very different CRISPR arrays.

Matches between independently acquired spacers from isolates with different CRISPR arrays demonstrates that isolates share a common viral pool from which they are independently infected. When spacers are compared to *Sulfolobus* viruses, plasmids, and *S. islandicus* genomes, there are two viruses (SSV) that are matched 100% by different spacers from different isolates. One virus has two spacers that match it while the other has three spacers. Just like the overlapping spacer matches, the spacers that match the same virus represent independent acquisitions, by different isolates, of the same virus. It is unlikely that the high frequency of spacer matches is due to a particular, rare sequence on the genome that is especially effective in resistance because we and others have shown that spacers are derived from throughout viral genomes [22], [32], [36], corresponding only to a short protospacer-associated motif (PAM), shown to be a dinucleotide sequence in *Sulfolobus* [35]. Therefore, the number of matches between spacers results from a combination of the selective force of virus-host interactions and/or the possibly low complexity of the viral community.

## Discussion

Our results show a significant amount of diversity in a population of *S. islandicus* from a single hot spring. This diversity is underestimated by MLSA sequences, but is revealed by CRISPR spacers present in these strains. Relationships described by MLSA are quite different from those observed using CRISPR locus sequences supporting the rapid evolution of CRISPR sequences relative to the rest of the genome. In addition, the population structure revealed by the individual-level of resolution provided by CRISPRs shows no evidence of a selective sweep or an epidemic structure in which one set of sequences is at high abundance relative to the rest of the population. Without a dominant clone, this snapshot of diversity within a single population is unlikely to follow a simple predator-prey model in which there are oscillations in strain abundance dependent upon resistance.

This is in contrast to the metagenome study of the clonal *Leptospirillum sp*, in which evidence of selective sweeps were identified in shared spacer sequences at the trailer end of the repeat spacer region of the locus as well as surrounding genes [25]. Explanations for the diversity and lack of strain dominance observed in the CRISPR loci in the *Sulfolobus* population include: 1) the addition of spacers in the bacterial population are slower, leaving time for a selective sweep to occur, 2) viruses in the *Leptospirillum* population are more virulent, causing a stronger selection, 3) the two studies have observed dynamics at different times during oscillations within populations, 4) there are differences in the number of interactions between strains in the highly structured biofilm and well mixed hot spring environments, and 5) there is a difference in recombination frequency between *Leptospirillum* and *Sulfolobus* that preserves the diversity of both genotype and CRISPR arrays in the *S. islandicus* population. In addition, in the metagenome study, as opposed to this study of isolates, it is difficult to link individual spacers within a CRISPR array and to link these arrays to very similar specific genotypes.

Spatial substructure would physically isolate hosts and/or virus populations from one another, allowing aggregate diversity to persist [37]. The demonstration that *S. islandicus* isolates from this pool are recombining suggests that they are not completely isolated from one another [28]. Also, independently acquired CRISPR spacer matches to the same mobile element indicate that individuals in this population share a pool of viruses and plasmids. Together, these data suggest that spatial structure within a single pool is not promoting the diversity in the CRISPR sequences we observe.

Theoretical and experimental studies have demonstrated that diversity of coexisting strains can arise in a population due to tradeoffs and variation in resistance phenotypes, and their costs are often associated with the efficiency of nutrient uptake [1], [13], [38]. Although the cost of CRISPR immunity has not been explicitly tested, it is unlikely that there is variation in the cost associated with spacer-specific resistance, because expression of the entire CRISPR locus occurs constitutively regardless of a match to an invading element [35]. Therefore, in the absence of an infecting virus, the cost of maintaining the CRISPR system is unlikely to be virus specific, and the cost of using the CRISPR system is not expected to vary across all members of the population that maintain similar numbers of CRISPR arrays [11]. Without variation in the cost of resistance, these models for the maintenance of diversity are also difficult to apply to this population.

We propose that CRISPR diversity may be maintained within this *S. islandicus* population due to clonal interference among individuals that have independently acquired resistance to viruses in their CRISPR loci. Different clones, each with a different CRISPR spacer to the same virus, compete with one another and therefore prevent a sweep that would purge all diversity from the environment [39]. Diversity is maintained in microbial populations because rapid, independent acquisition of resistance by different genotypic backgrounds prevents periodic selective sweeps of resistant types. The evolution of the CRISPR locus through spacer addition is rapid enough that multiple strains within the same population can easily acquire the same resistance to a dominant virus. Each uniquely acquired CRISPR spacer is present in the population at a different frequency due to the timing at which the resistance was originally gained [40].

This is conceptually similar to the theoretical model described by Rodriguez-Valera et al. in which diversity is maintained due to the rapid evolution of virus receptors in genomic islands [6], however it provides a mechanism for the rapid generation of variation that directly results from virus infection (CRISPR spacer acquisition) and is therefore dependent on viral density. Also, addition of new CRISPR spacers provides a mechanism of resistance where there is little potential for variation in cost of resistance. Finally, using CRISPR spacers to assess population dynamics allows direct linkage between viruses and resistance profiles which does not rely on inferring the importance or expression of cell surface proteins. It should be noted however, that our focus on MLSA of shared core gene markers and CRISPR sequences prohibits assessment of variation in other resistance mechanisms in *S. islandicus* that may play an additional role in the maintenance of diversity within this population.

The rapid acquisition of independent CRISPR spacers consistent with our model has been shown by Barrangou et al. in laboratory infections of *Streptococcus* sp. In that study, when the host is challenged by one virus, multiple resistant hosts are found, each with different spacers that give immunity to the same virus [17]. Therefore, when differing immunities to the same virus are present in a population, one virus would not be able to cause a sweep of a single resistant genotype in the population that would result in a loss of diversity. Our data shows that independently acquired CRISPR spacers match one another and presumably the same virus, supporting the idea that resistance to the same virus occurs independently in different strains in the same population. Since most of these spacer to spacer matches are between strains that are not related by CRISPR spacer arrays, CRISPR spacers, far from promoting sweeps that remove diversity, actually promote diversification among strains within a population.

Our proposal that diversity is maintained through clonal interference among independently acquired CRISPR variants depends upon there being a fitness advantage to resistance and consequent cost of viral infection. However, both lytic and non-lytic viruses infect *Sulfolobus* species [41], [42]. Therefore, in order to understand how microbial diversity is shaped by CRISPR immunity, it will be important to consider the diverse array of virus-host interactions when developing future models.

## Methods

### Strain isolation and DNA extraction

*Sulfolobus islandicus* strains from hot spring M16, located in the Mutnovsky Volcano region of Kamchatka, Russia were isolated and DNA was extracted as in Whitaker et al. [43]. Two *S. islandicus* strains were previously isolated and sequenced [33]. Thirty-seven additional isolates from the M16 pool, previously described as hot spring B, were isolated, thirty-one of which were used in the previous study [28]. All strains went through three additional rounds of colony purification on solid media to ensure purity. Seven slightly different methods of isolation were used on these strains (see Table S2), however ANOVA does not find any significant difference in MLSA types from each type of isolation (p = 0.13).

### MLSA

MLSA loci and primer sequences are listed in Table S3. Loci were selected from *S. islandicus* core genes [33] to be evenly distributed around the genome and to maximize SNPs in the Mutnovsky genomes. All loci were amplified by PCR in 28 ul reactions with 6 uL 5x Green GoTaq Reaction Buffer (Promega), 2 uL 25 mM MgCl_2_, 0.14 uL 0.2 mM dNTP, 0.5 uL of each 10 uM primer, and 0.14 uL 5 u/uL GoTaq DNA Polymerase (Promega). PCR conditions for all loci were as follows: 94°C for 5 min, 30 cycles of 94°C 30 sec, annealing temperature (Table S3) 75 sec, 72°C 90 sec 30 sec, and a final incubation at 72°C for 5 min. PCR products were sequenced with the forward primer at the Core DNA Sequencing Laboratory (Roy J. Carver Biotechnology Center, University of Illinois at Urbana-Champaign). Sequences were deposited in GenBank, and accession numbers (HQ123504-HQ123546) are listed in Table S4. Nucleotide sequences for the MLSA markers were automatically aligned with T-coffee [44] and manually inspected with MacClade [45]. The phylogeny was inferred using a concatenated (all loci) alignments under Maximum Parsimony with PAUP* 4.0b10 [46]. Heuristic search was performed by 10 random addition sequence replicates. Non-parametric bootstrapping [47] was conducted with 1000 replicates of 10 random addition sequence replicates. Unique alleles were assigned to sequences that contain one or more nucleotide polymorphisms from the dominant allele. Recombination to mutation ratio (r/m) was estimated using a model of coalescence with gene conversion implemented in the Clonal Frame software V.1.1 [30]. r/m values were taken from the convergence values of two runs of 250000 iterations each with a burnin chain of 100000 iterations.

### CRISPR PCR Amplification

Primers to amplify the CRISPR loci were designed by genomic comparison of the CRISPR region of strains of *S. islandicus* from the Mutnovsky Volcano region of Kamchatka, Russia [32] and recently sequenced genomes (unpublished data). These primers are AB1f (5′TCCCGGGTTTAGTAGGGAGT GAAA), AB1r (5′CCATACGGCTTCCCTAGATTTAGATT), A1.2r (5′CATCAACAGTTAGCGGAAGTGAGG), A1.2f (5′GGGAGGTAGGGTGTTGTCCTAAA), ABrU (5′TCCCACCCTCATGCTGGAATTCTT), and 16.43.AB1r (5′GGAATGGGAATTGCTGAAATAGCG) to amplify the AB1 locus. Primers AB2f (5 CTAGTTGCTTCCATTAAGTCGCTC), AB2r (5′TCCCGGGTTTAGTAGGGAGTGAAA), A2.2f (5′TGCCTTGTCTCATTAATGCGCGG), and A2.2r (5′GGGAGGTAGGGTGTTGTCCTAAA) were designed to amplify the AB2 locus. CDr (5′CGGTCACATGAGGAGTAAAGGA), CDf (5′CGTCCCATCACTTGCTTTGAGCAT), CDf3 (5′TTGAATGAGGCTTACCGGAAGGGA), and CDr3 (5′TTAGGCCCAGAAGGGAACCATCAA) were designed to amplify the CD locus.

All loci were amplified by PCR in 20 ul reactions with 4 ul 5x Phusion HF Buffer, 200 uM dNTP, 0.5 uM primer, and 0.02 U/ul Phusion DNA Polymerase (Finnzymes). PCR conditions for all loci were as follows: 98°C for 30 sec, 30 cycles of 98°C 30 sec, annealing temperature (depending on primer set) 10 sec, 72°C 2 min 30 sec, and a final incubation at 72°C for 5 min. The various primer sets amplified at the following annealing temperatures: AB1f/AB1r at 57°C, AB1f/16.43.AB1r at 57.5°C, AB1f/ABrU at 64°C, A1.2f/A1.2r at 59.2°C, AB2f/AB2r at 53–57°C, A2.2f/A2.2r at 58.5°C, CDf/CDr at 51–57°C, CDf3/CDr at 55°C, and CDf3/CDr3 at 54°C. PCR products were sequenced with both forward and reverse primers at the Core DNA Sequencing Laboratory (Roy J. Carver Biotechnology Center, University of Illinois at Urbana-Champaign). Sequences were deposited in GenBank, and accession numbers (HQ198372-HQ198558) are listed in Table S5.

### CRISPR spacer identification and comparison

CRISPR PCR products were sequenced as with MLSA amplicons and manually trimmed and checked for sequencing errors using Sequencher 4.9 (Gene Codes, Ann Arbor, MI, USA). Individual spacers were removed from the sequences by manually extracting the sequence between the repeats sequences: A repeat is GATAATCTACTATAGAATTGAAAG and C repeat is GATTAATCCTAAAAGGAATTGAAAG. Spacers were grouped according to the ends of the loci they came from, and BLASTn [48] with E*<*0.001 was used to find 100% spacer matches. Strains were grouped within each locus as being ancestrally related if spacers in the array matched each other in the same spacer context (multiple identical spacer matches in a row, allowing for some spacer loss). The results are interpreted manually and shown in Figure 2 by colored boxes and vertically aligned ancestrally identical spacers. Spacers were compared to one another for non-ancestral matches in Sequencher 4.9. Assembly parameters of 88% minimum match with 17 nt minimum overlap were used to define unique spacers and resulting contigs were spacer-spacer matches. This allows a maximum of 4 SNPs per pair.

Spacers were compared to a database of *Sulfolobus* genome, virus, and plasmid sequences. This database included all *Sulfolobus* genome, virus, and plasmid sequences found on the Sulfolobus Database (http://dac.molbio.ku.dk/dbs/Sulfolobus/cbin/mutagen.pl –01/01/10), plus the *Sulfolobus islandicus* genomes of L.S.2.15, L.D.8.5, M.14.25, M.16.4, M.16.27, Y.G.57.14, Y.N.15.51 [33], and U.3.28 (http://www.jgi.doe.gov). Spacers were blasted against this database using BLASTn with parameters *r* = 1, *q* = −1, *G* = −4 and significant matches were E*<*0.001.

### Rarefaction

Rarefaction curves were constructed using Mothur [49] with default parameters. The MLSA rarefaction curve was constructed with the same concatenated nucleotide alignment used for phylogeny, while the CRISPR rarefaction curve was constructed based on the colored representation of the CRISPR loci spacer arrays. Spacers in each vertical position were given a letter code to represent the color, so that identical spacers in each column had the same code, which differed from the code given to different spacers in that column.

## Supporting Information

Figure S1 Rarefaction curves of MLSA and CRISPR sequences. Rarefaction curves of (A) the concatenated nucleotide alignment of 12 MLSA loci and (B) the concatenated coded CRISPR spacer arrays from 39 *S. islandicus* isolates from a single hot spring. The number of isolates (X-axis) is plotted against the number of OTUs (Y-axis) determined by the level of divergence for each line (for A, unique, distance <.0049, and distance of 0.01 and for B, unique, distance of 0.05, and distance of 0.5).
(0.62 MB TIF)

Figure S2 Primer design and implementation. Primer design schematic (A) and table of primer sets and temperatures used for each strain at each CRISPR locus (B). In (A), primers are shown by their position on the reference genome M.16.27, with the head of the arrow matching the 5′ end of the primer. Arrows above the schematic indicate the approximate location of primers on M.16.27 while arrows below the schematic indicate primers designed on other fully sequenced genomes or PCR products that do not match sequence in M.16.27. In (B), the primer pair used for each locus is listed, with annealing temperature used if multiple temperatures are used for that primer pair. ‘X’ and ‘NA’ as in Figure 1. Sequenced refers to those strains that were fully sequenced and CRISPR spacer sequences were determined without PCR.
(0.57 MB TIF)

Table S1 Pairs of independently acquired spacers match the same virus or plasmid. Each spacer pair is numbered and spacer names are given as an isolate number followed by a locus position number as in Figure 2. In the case of a spacer being ancestrally identical to other spacers, the first (top) spacer from Figure 2 is listed here, though all spacers have a • in Figure 2. * indicates spacer pair from isolates with similar CRISPR arrays.
(0.30 MB TIF)

Table S2 Isolation methods for *S. islandicus* isolates. Isolates are listed with their isolation method. There are seven different isolation methods that yielded colonies: DT (dextrin and tryptone) spread plate as described in [43]; DT overlay plate containing DT media plus an overlay of 0.002% Gelrite (Sigma), 0.002% K2SO4, and 0.002% L-glutamic acid; DTS spread plate containing standard DT media plus an overlay of 0.006% Gelrite and 0.002% colloidal sulfur; DTS overlay plate containing DTS plus additional overlay described above. 1∶50 indicates a 1∶50 dilution of sample prior to plating.
(0.33 MB TIF)

Table S3 MLSA primers. MLSA loci and primers listed with annealing temperature (T) and length of amplicon. * indicates loci used in [28].
(0.52 MB TIF)

Table S4 MLSA sequence allele accession numbers. Allele numbers for each of the 12 MLSA loci for each strain are shown in the table on the left. Locus marker codes correspond to Table S3. MLSA loci are listed with each allele and accession number in the table on the right.
(0.67 MB TIF)

Table S5 CRISPR loci accession numbers. Accession numbers for CRISPRs are listed by strain and locus. The first number at each locus corresponds to the leader end sequence and the second number corresponds to the trailer end. ‘NA’ and ‘X’ as in Figure 1. The CRISPR_id from the CRISPRdb website [50] is shown for M.16.27 and M.16.4.
(0.82 MB TIF)
