# Supplementary figures and images for: CRISPR Associated Diversity within a Population of Sulfolobus islandicus
Source: PLoS One. 2010 Sep 28;5(9):e12988. doi: 10.1371/journal.pone.0012988 (PMC2946923; doi:10.1371/journal.pone.0012988)

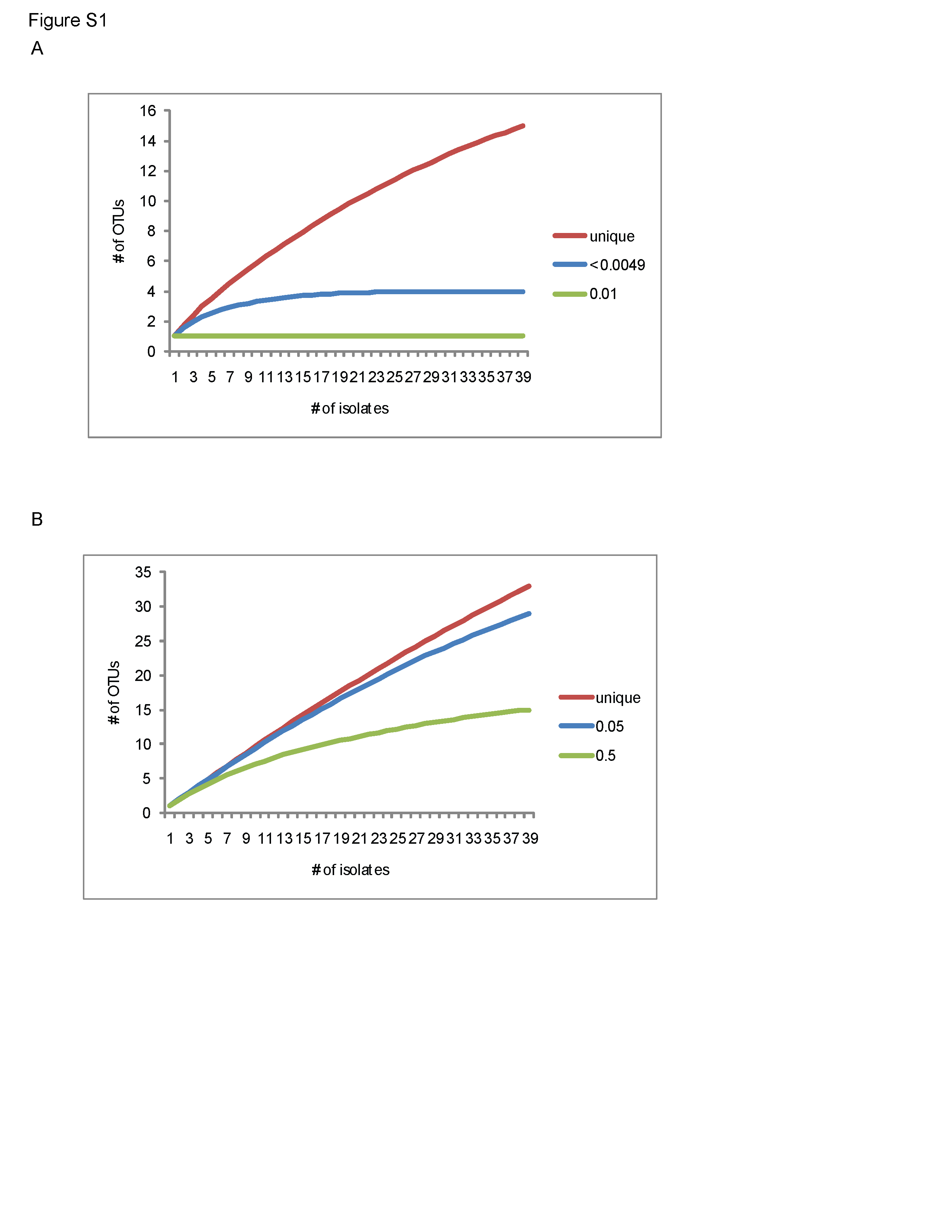

Supplement: Figure S1 — Rarefaction curves of MLSA and CRISPR sequences. Rarefaction curves of (A) the concatenated nucleotide alignment of 12 MLSA loci and (B) the concatenated coded CRISPR spacer arrays from 39 S. islandicus isolates from a single hot spring. The number of isolates (X-axis) is plotted against the number of OTUs (Y-axis) determined by the level of divergence for each line (for A, unique, distance <.0049, and distance of 0.01 and for B, unique, distance of 0.05, and distance of 0.5). (0.62 MB TIF) [file pone.0012988.s001.tif]

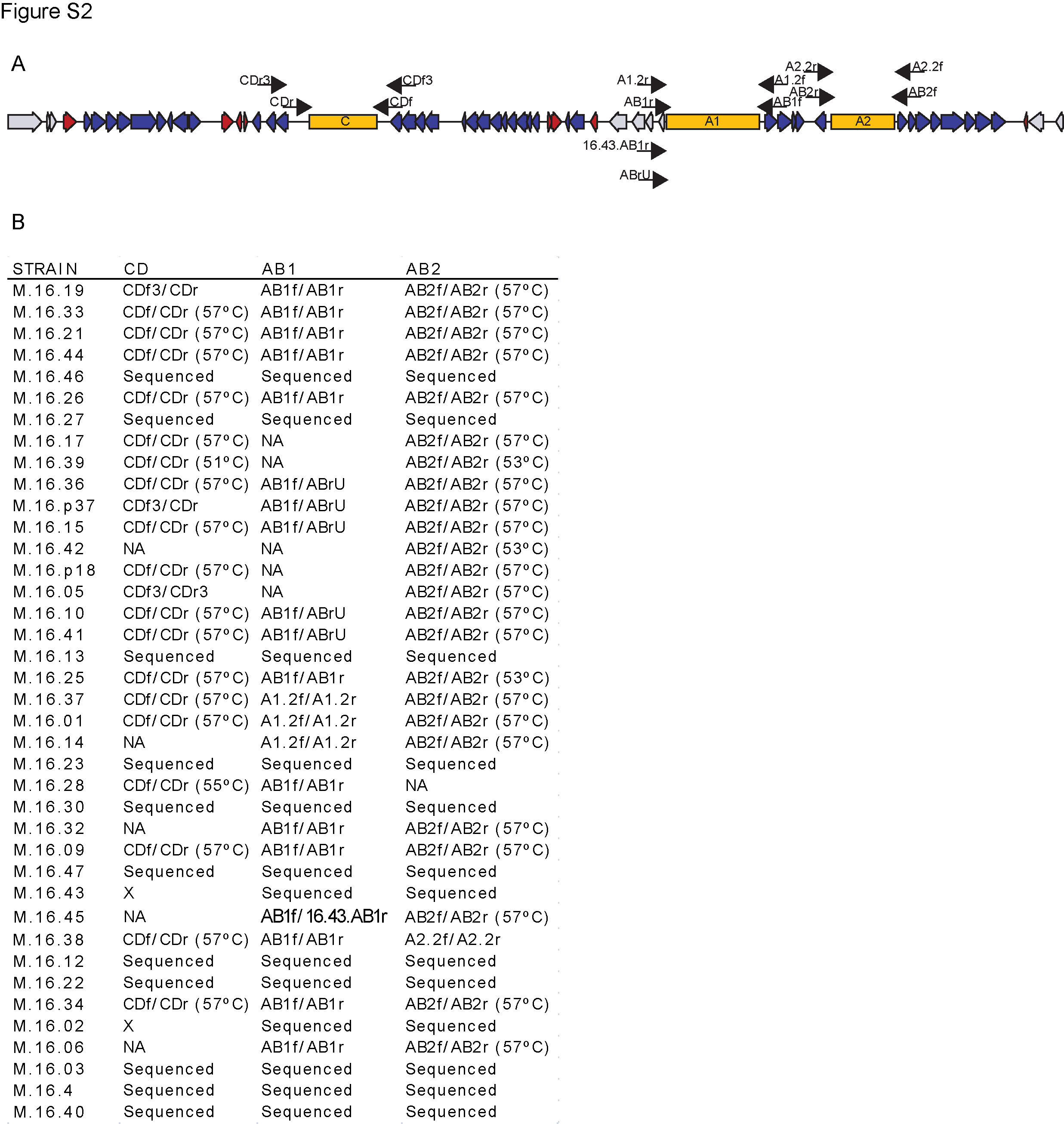

Supplement: Figure S2 — Primer design and implementation. Primer design schematic (A) and table of primer sets and temperatures used for each strain at each CRISPR locus (B). In (A), primers are shown by their position on the reference genome M.16.27, with the head of the arrow matching the 5′ end of the primer. Arrows above the schematic indicate the approximate location of primers on M.16.27 while arrows below the schematic indicate primers designed on other fully sequenced genomes or PCR products that do not match sequence in M.16.27. In (B), the primer pair used for each locus is listed, with annealing temperature used if multiple temperatures are used for that primer pair. ‘X’ and ‘NA’ as in Figure 1. Sequenced refers to those strains that were fully sequenced and CRISPR spacer sequences were determined without PCR. (0.57 MB TIF) [file pone.0012988.s002.tif]

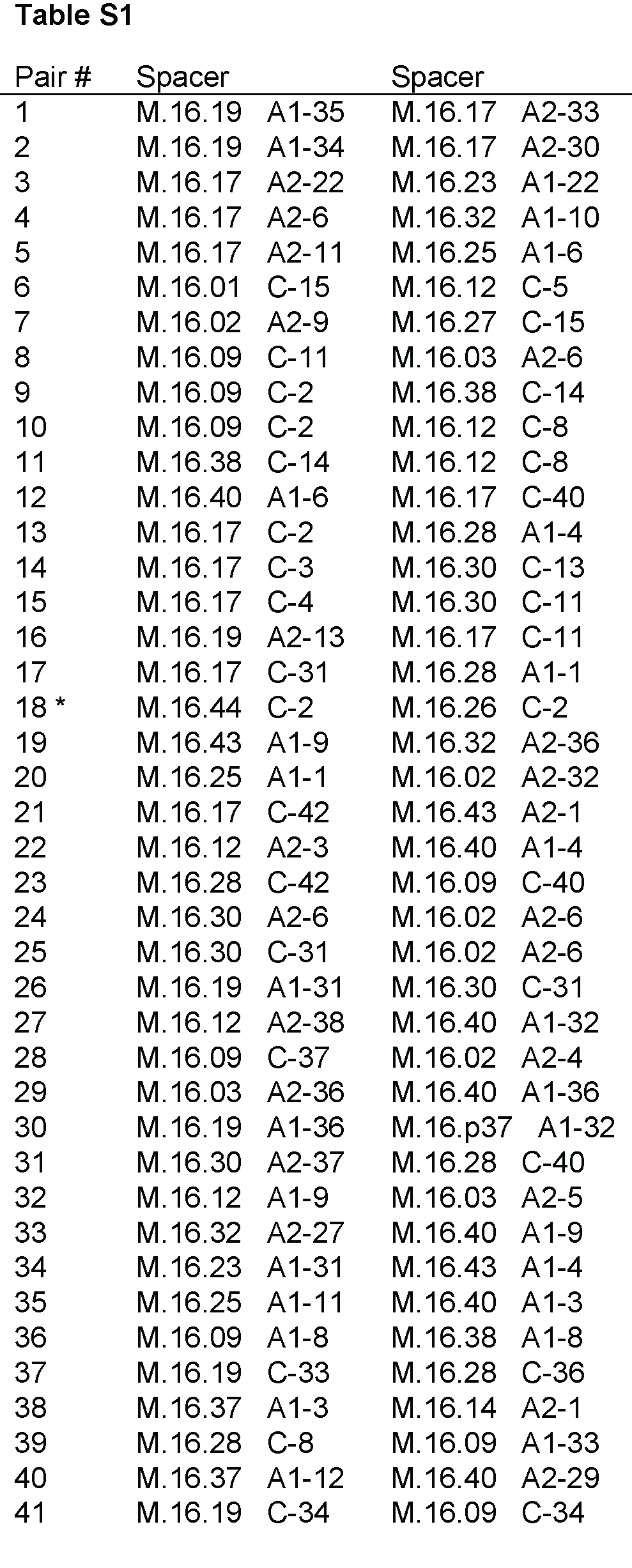

Supplement: Table S1 — Pairs of independently acquired spacers match the same virus or plasmid. Each spacer pair is numbered and spacer names are given as an isolate number followed by a locus position number as in Figure 2. In the case of a spacer being ancestrally identical to other spacers, the first (top) spacer from Figure 2 is listed here, though all spacers have a • in Figure 2. * indicates spacer pair from isolates with similar CRISPR arrays. (0.30 MB TIF) [file pone.0012988.s003.tif]

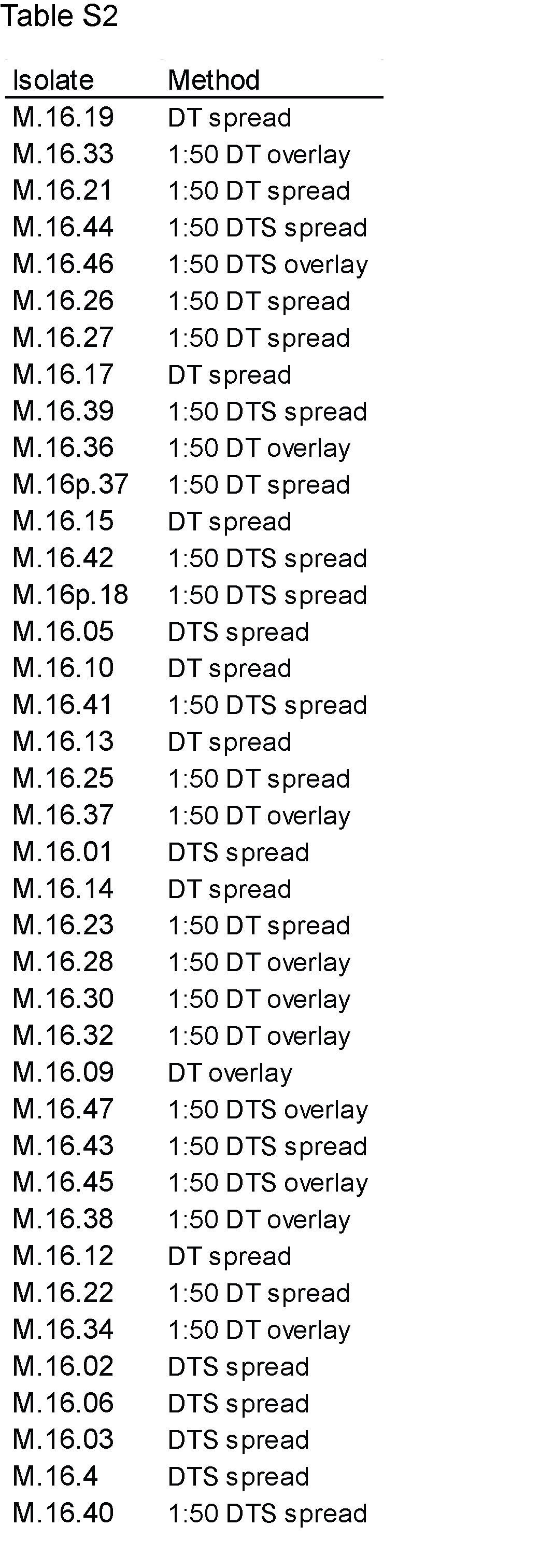

Supplement: Table S2 — Isolation methods for S. islandicus isolates. Isolates are listed with their isolation method. There are seven different isolation methods that yielded colonies: DT (dextrin and tryptone) spread plate as described in [43]; DT overlay plate containing DT media plus an overlay of 0.002% Gelrite (Sigma), 0.002% K2SO4, and 0.002% L-glutamic acid; DTS spread plate containing standard DT media plus an overlay of 0.006% Gelrite and 0.002% colloidal sulfur; DTS overlay plate containing DTS plus additional overlay described above. 1∶50 indicates a 1∶50 dilution of sample prior to plating. (0.33 MB TIF) [file pone.0012988.s004.tif]

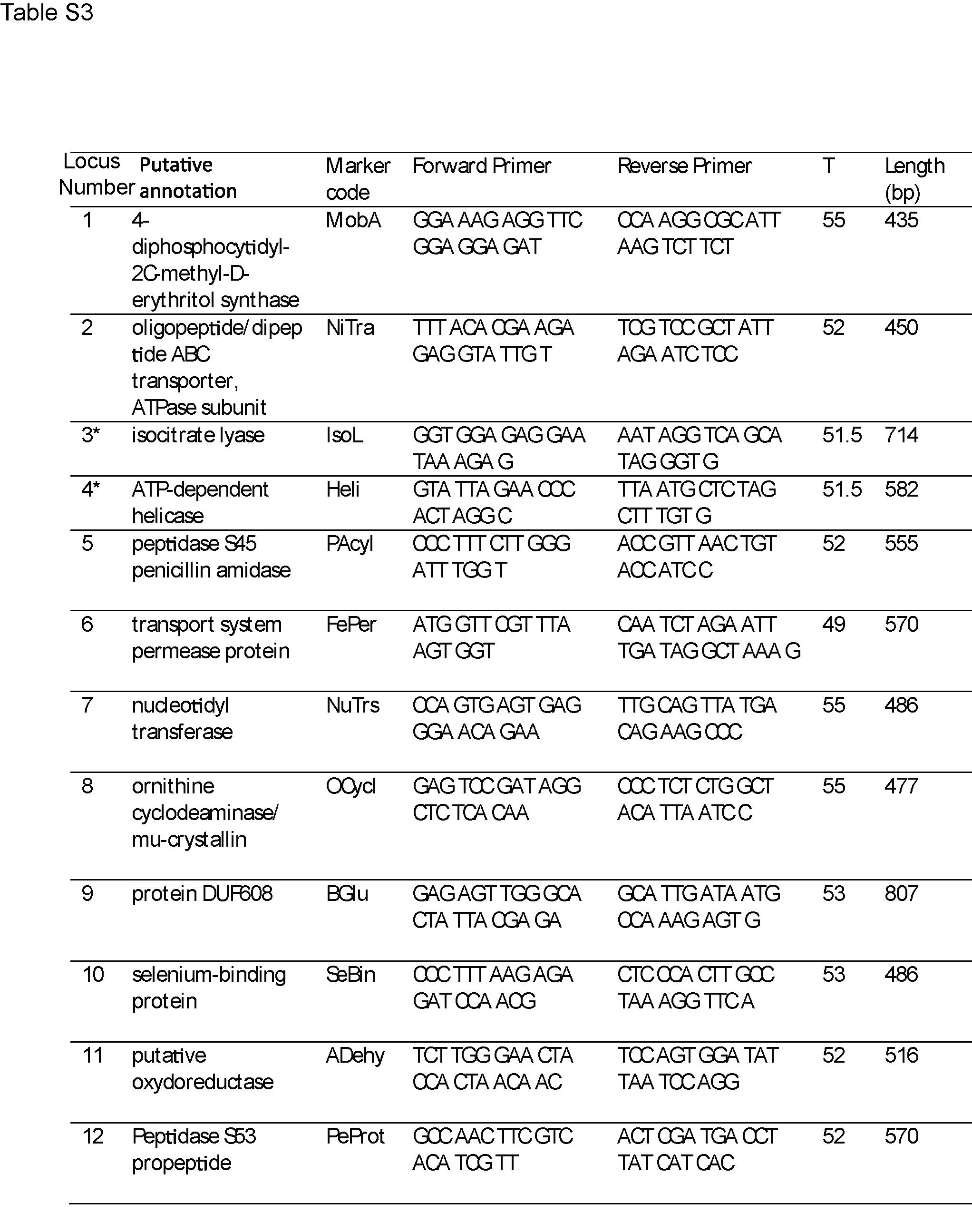

Supplement: Table S3 — MLSA primers. MLSA loci and primers listed with annealing temperature (T) and length of amplicon. * indicates loci used in [28]. (0.52 MB TIF) [file pone.0012988.s005.tif]

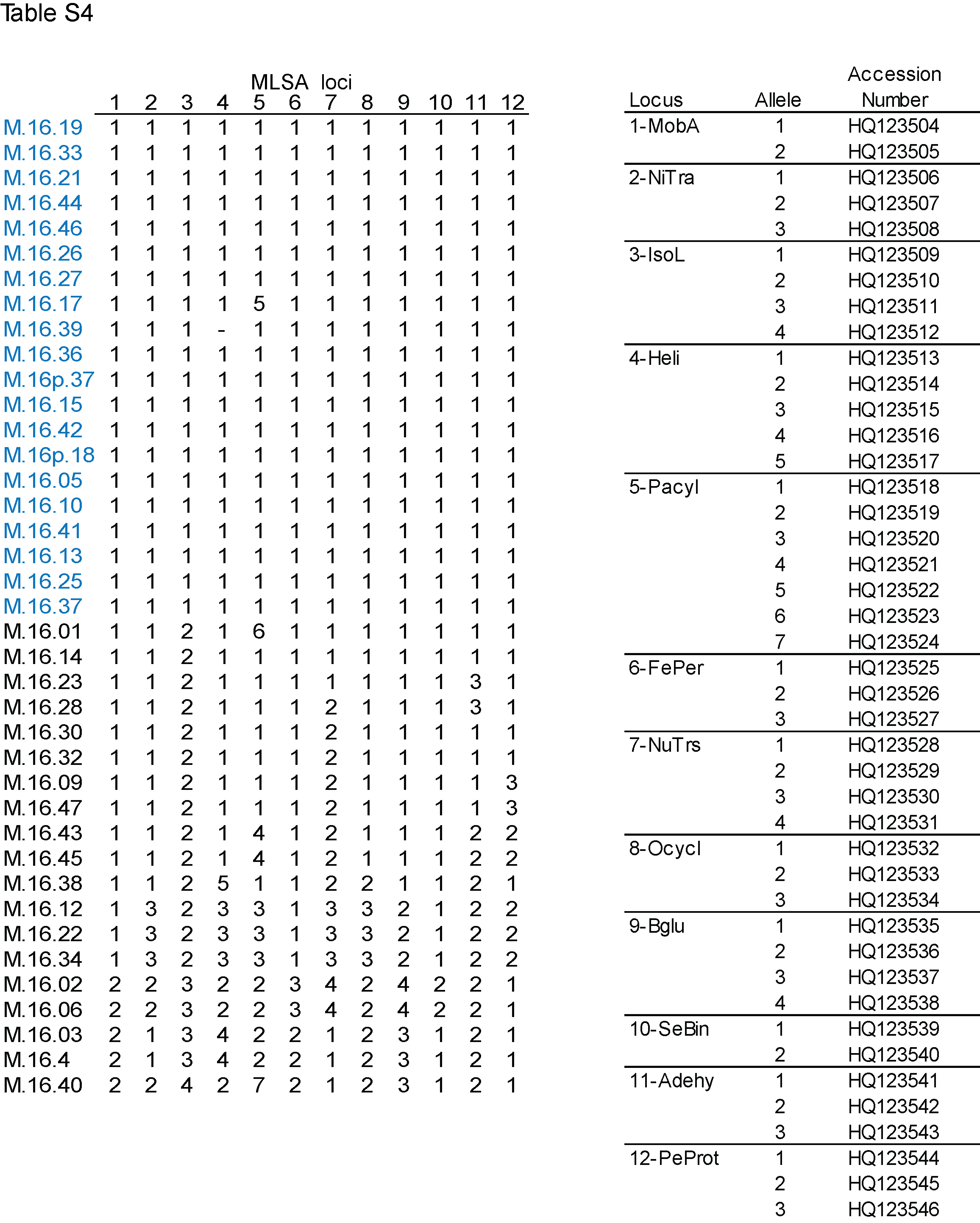

Supplement: Table S4 — MLSA sequence allele accession numbers. Allele numbers for each of the 12 MLSA loci for each strain are shown in the table on the left. Locus marker codes correspond to Table S3. MLSA loci are listed with each allele and accession number in the table on the right. (0.67 MB TIF) [file pone.0012988.s006.tif]

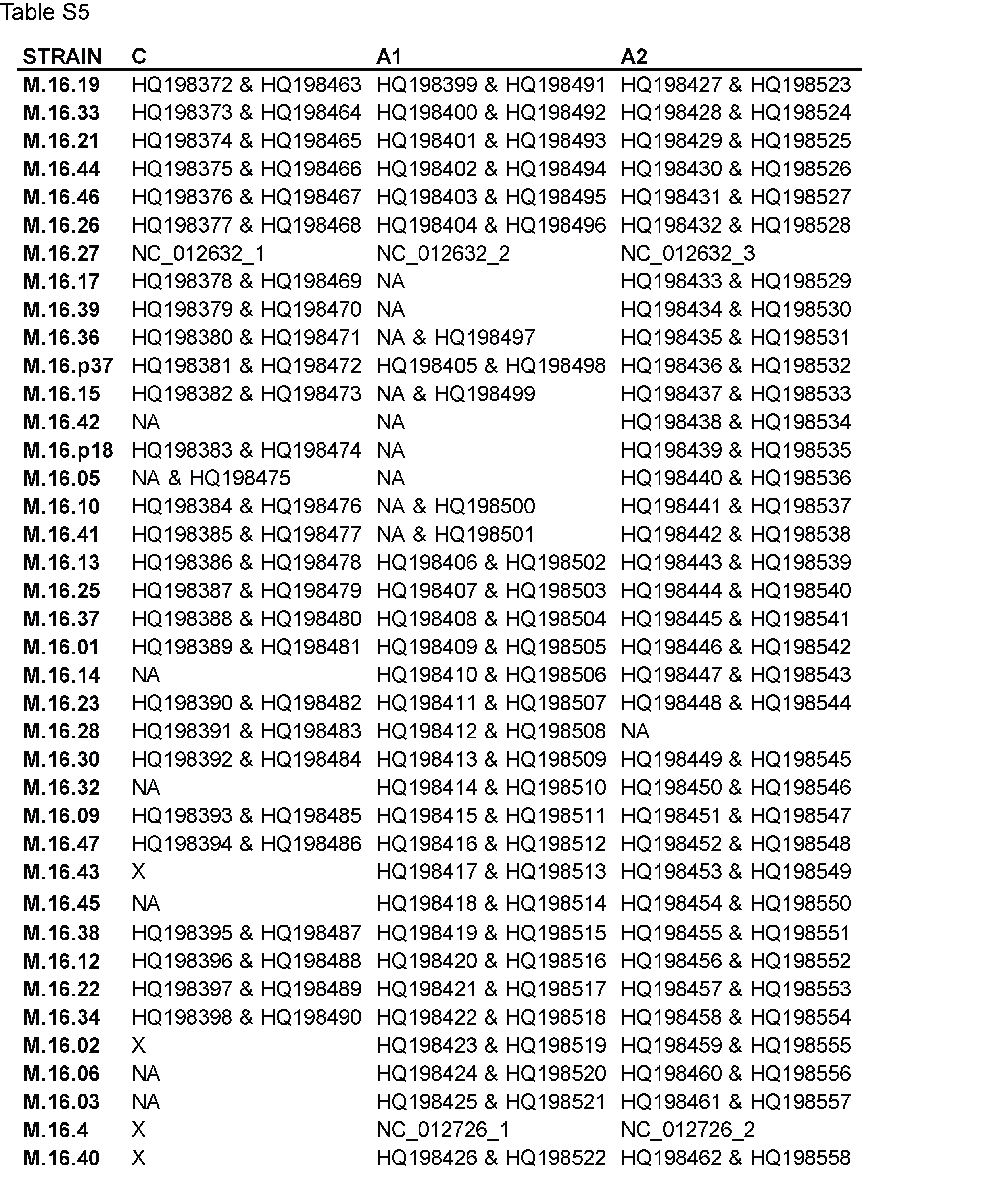

Supplement: Table S5 — CRISPR loci accession numbers. Accession numbers for CRISPRs are listed by strain and locus. The first number at each locus corresponds to the leader end sequence and the second number corresponds to the trailer end. ‘NA’ and ‘X’ as in Figure 1. The CRISPR_id from the CRISPRdb website [50] is shown for M.16.27 and M.16.4. (0.82 MB TIF) [file pone.0012988.s007.tif]
